# CFTR Rescue in Intestinal Organoids with GLPG/ABBV-2737, ABBV/GLPG-2222 and ABBV/GLPG-2451 Triple Therapy

**DOI:** 10.3389/fmolb.2021.698358

**Published:** 2021-09-15

**Authors:** Eyleen de Poel, Sacha Spelier, Ricardo Korporaal, Ka Wai Lai, Sylvia F. Boj, Katja Conrath, Cornelis K. van der Ent, Jeffrey M. Beekman

**Affiliations:** ^1^ Department of Pediatric Respiratory Medicine, Wilhelmina Children’s Hospital, University Medical Center, Utrecht University, Utrecht, Netherlands; ^2^ Regenerative Medicine Utrecht, University Medical Center, Utrecht University, Utrecht, Netherlands; ^3^ Hubrecht Organoid Technology (HUB), Utrecht, Netherlands; ^4^ Galapagos NV, Mechelen, Belgium

**Keywords:** cystic fibrosis, CFTR, F508del, forskolin induced swelling assay, intestinal organoids, personalized medicine, CFTR modulator therapy

## Abstract

Cystic fibrosis transmembrane conductance regulator (CFTR) modulators have transformed the treatment of cystic fibrosis (CF) by targeting the basis of the disease. In particular, treatment regimen consisting of multiple compounds with complementary mechanisms of action have been shown to result in optimal efficacy. Here, we assessed the efficacy of combinations of the CFTR modulators ABBV/GLPG-2222, GLPG/ABBV-2737 and ABBV/GLPG-2451, and compared it to VX-770/VX-809 in 28 organoid lines heterozygous for F508del allele and a class I mutation and seven homozygous F508del organoid lines. The combination ABBV/GLPG-2222/ABBV-2737/ABBV/GLPG-2451 showed increased efficacy over VX-770/VX-809 for most organoids, despite considerable variation in efficacy between the different organoid cultures. These differences in CFTR restoration between organoids with comparable genotypes underline the relevance of continuing to optimize the ABBV/GLPG‐Triple therapy, as well as the in vitro characterization of efficacy in clinically relevant models.

## Background

Cystic fibrosis (CF) is a monogenetic, autosomal, recessive disease caused by mutations in the cystic fibrosis transmembrane conductance regulator (CFTR) gene (Sosnay et al., 2013). Various mutations in CFTR have been characterized that result in dysfunction or complete absence of CFTR, which is followed by ion misbalance and subsequent aberrant fluid secretion in multiple organ systems such as the intestine, airways and pancreas (Sosnay et al., 2013). For patients with common mutations, like F508del and G5551D multiple CFTR modulating compounds have been developed that rescue the mutation specific defects. These first generation CFTR modulating drugs however do not restore CFTR function in CF patients with one F508del mutation sufficiently (Rowe et al., 2017). In line with these clinical observations, *in vitro* experiments still detect the presence of immature (B-band) CFTR after treatment with CFTR corrector VX-809 treated F508del cells (Van Goor et al., 2011), providing the rationale for combining two correctors with a complementary mode of action that collectively further restore the trafficking defect. The recent discovery of the triple combination of VX-445/VX-661/VX-770 clearly shows that indeed a combination of two correctors with a potentiator is required to obtain high efficacy CFTR modulation (Veit et al., 2020). However, recent clinical studies showed great variation in triple treatment efficacy, highlighting the need for expanding the pool of treatment options (Davies et al., 2018). One such new triple therapy that showed effective rescue of CFTR in human bronchial epithelial cells is developed by Abbvie and consists of the Abbvie/GLPG correctors ABBV/GLPG-2222 (Wang et al., 2018) and GLPG/ABBV-2737 (de Wilde et al., 2019) and potentiator ABBV/GLPG-2451 (Van der Plas et al., 2451). ABBV/GLPG-2222 and ABBV/GLPG-2451 exhibit similarities in biological activity with respectively VX-809 and VX-770, but rescue F508del-CFTR more potently (Singh et al., 2016). In an effort to further increase the efficacy of the combination of ABBV/GLPG-2222/ABBV/GLPG-2451, another corrector with a complementary mode of action, termed GLPG/ABBV-2737 was developed that exerted functional synergy with ABBV/GLPG-2222 and VX-809 (de Wilde et al., 2019). Combining these modulators into a triple therapy resulted in a two-fold increase in Cl^−^ current in F508del/F508del HBE cells compared to VX770/VX809 treatment (Wang et al., 2018).

In this report we compare the efficacy of single, dual or triple combinations of ABBV/GLPG-2222, GLPG/ABBV-2737 and ABBV/GLPG-2451 to VX-809/VX-770 using intestinal organoids and the forskolin (FSK) induced swelling (FIS) assay (Boj et al., 2017). *In vitro* FIS response of patient-derived intestinal organoids upon modulator therapy has been shown to predict *in vivo* response to therapy (Dekkers et al., 2016; Berkers et al., 2019), making this model a relevant model in the context of preclinical drug discovery and lead selection. To assess efficacy and between-patient variability of the ABBV/GLPG-compounds on rescuing F508del-CFTR, we measured FIS in 35 intestinal organoid cultures, seven expressing F508del/F508del-CFTR and 28 expressing F508del/minimal function CFTR. All organoids cultures did not exhibit swelling when exposed to solely forskolin, indicating the absence of residual CFTR function for all organoid cultures. Ultimately, the aim of this report is to assess the preclinical efficacy of a newly developed triple therapy and to identify people with CF likely to benefit from modulator therapy.

## Rescue of minimal-function and residual-function CFTR mutations with ABBV/GLPG-2222, GLPG/ABBV-2737 and ABBV/GLPG-2451

**FIGURE 1:**
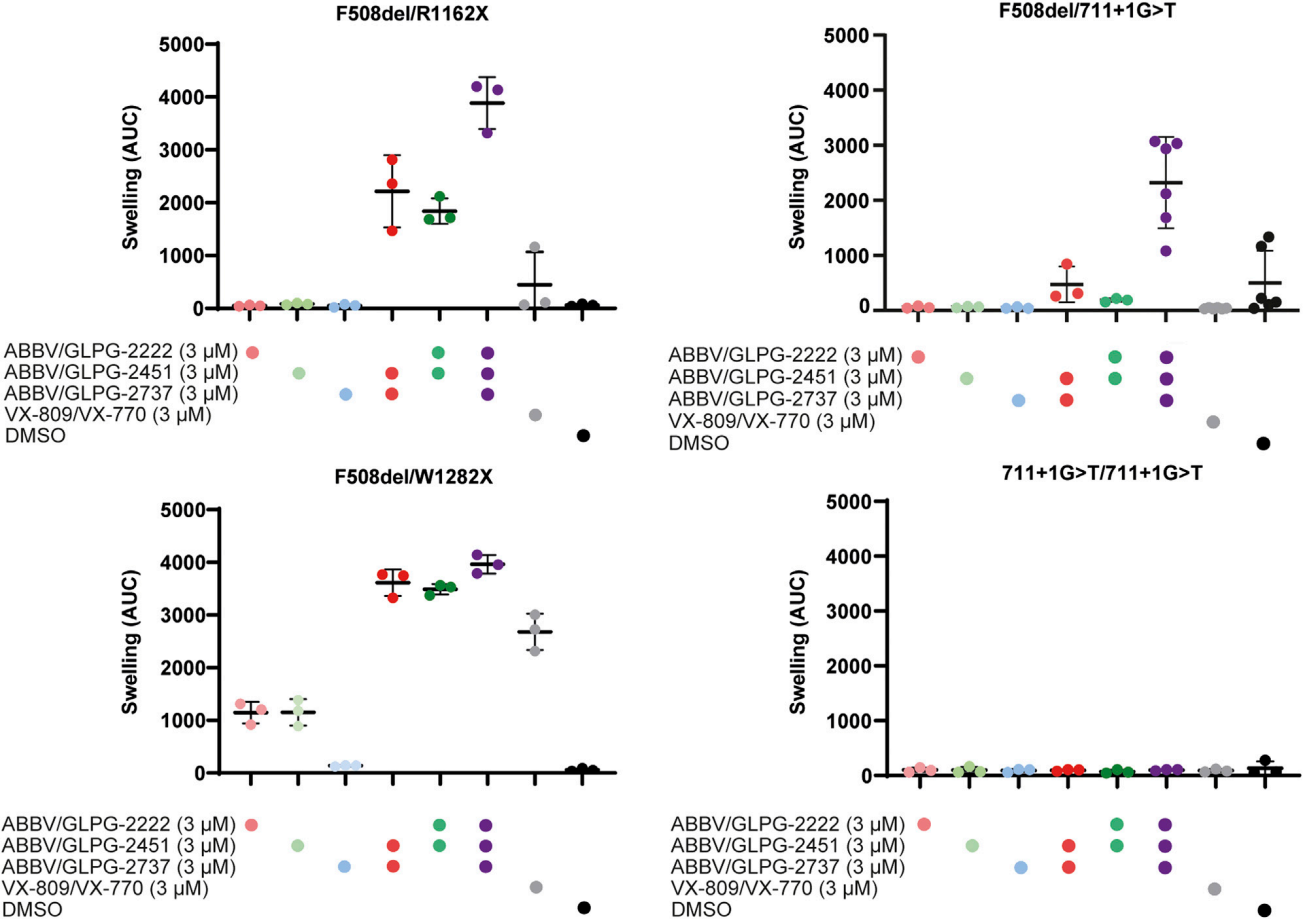
CFTR function rescue with single, dual and triple ABBV/GLPG modulator therapy in intestinal organoids. FIS responses of three organoid cultures expressing F508del/minimal function genotypes and one organoid culture homozygous for 711+1G > T, stimulated with 5.0 µM forskolin for 1h. FIS responses were measured upon treatment with ABBV/GLPG compounds, VX-809/VX-770 and DMSO. n = 3 or n = 6, each bar represents mean ± SD.

New therapies under development for F508del should be sufficiently efficacious for people with a single F508del allele. For this reason, we first compared the efficacy of compounds on three organoid cultures with F508del in compound heterozygosity with established and characterized non-functional class I alleles so that impact of treatment on a single F508del was established (Figure 1, Supplementary Table 1). Whereas single compounds did not result in increased levels of organoid swelling in F508del/R1162X and F508del/711 + 1G > T, it resulted in a mild increase in swelling (± 1000 AUC) for the F508del/W1282x organoid culture. The two dual combinations of one corrector (ABBV/GLPG-2222 or GLPG/ABBV-2737) and the potentiator ABBV/GLPG-2451, resulted in substantial swelling. For two organoid cultures, expressing F508del/R1162X and F508del/711+1G > T CFTR, the combination of all three ABBV/GLPG compounds resulted in a further increased AUC. Interestingly, the combination of one corrector and ABBV/GLPG-2451 resulted in similar swelling levels as the ABBV/GLPG-Triple in the W1282X organoid culture.

**FIGURE 2:**
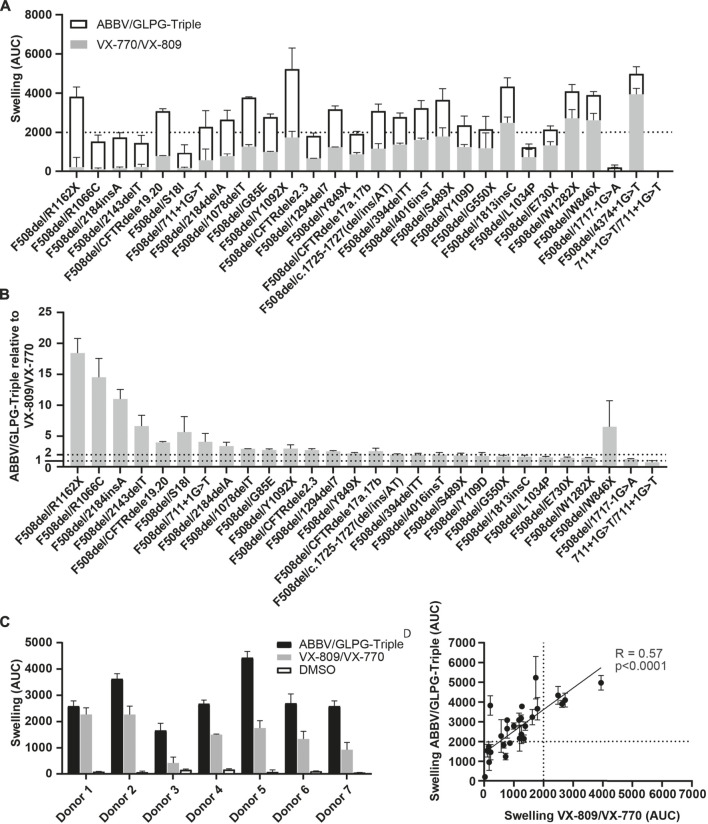
FIS response of F508del/minimal function organoid cultures upon ABBV/GLPG-Triple therapy. **(A)** FIS response upon VX-770/VX-809 (3 μM, gray bars) and Abbvie-Triple (ABBV/GLPG-2222 3 µM + GLPG/ABBV-2737 3 µM + ABBV/GLPG-2451 3 µM,white bars, stacked) treatment, corrected for the DMSO condition. n = 3, each bar represents mean + SD. CFTR correctors were added 24h prior to FIS measurements, CFTR potentiators were simultaniously added with 5 µM FSK. **(B)** FIS reponse upon the ABBV/GLPG-Triple (3 µM) relative to VX-770/VX-809 (3 µM) treatment, both corrected for the DMSO condition. n = 3, each bar represents mean + SD. Similar raw data was used in **(A and B)**, yet differentially illustrated. **(C)** FIS response of seven F508del/F508del organoid cultures upon treatment with ABBV/GLPG-Triple (ABBV/GLPG-2222 1 µM + GLPG/AB­ BV-2737 1 µM + ABBV/GLPG-2451 1 µM) or VX-809/VX-770 (1 µM) and 0.8 µM FSK. n = 3, bars represent mean + SD. **(D)** Correlation of FIS responses upon the ABBV/GLPG-Triple (3 µM) and VX-770/VX-809 (3 µM) treatment, n = 3, each datapoint represents mean ± SD.

Next, the ABBV/GLPG-Triple as well as VX-809/VX-770 were tested on 28 organoid cultures harboring F508del/minimal function genotypes (Figure 2A, Supplementary Table 1)**.** Upon exposure to the ABBV/GLPG-Triple substantial AUC values were observed (AUC >2000 for 19/28 lines). Although the efficacy of the triple therapy greatly varied between the different organoid cultures, organoid swelling increased on average by 297% with the ABBV/GLPG-Triple compared to VX-809/VX-770 (Figure 2B, Supplementary Table 1). Not only did we observe large variation in CFTR function rescue between organoids expressing distinct F508del/minimal function genotypes, we also observed variation in treatment response between organoids carrying F508del/F508del-CFTR (Figure 2C, Supplementary Table 1). Moreover, we compared Orkambi response and ABBV/GLPG-Triple response in the panel of 28 organoid lines and observed a linear positive correlation (R = 0.57) indicating that organoid cultures that respond to Orkambi are likely to regain CFTR when treated with the ABBV/GLPG-Triple (Figure 2D, Supplementary Table 1).

## Discussion

The objective of this study was to assess efficacy of CFTR-F508del restoration by the single ABBV/GLPG modulators (ABBV/GLPG-2222/GLPG/ABBV-2737/ABBV/GLPG-2451) and all combinations thereof in comparison to VX-809/VX-770 by measuring FIS in organoids carrying at least one single F508del allele. We observed highest CFTR-restoring efficacy with the ABBV/GLPG-Triple, which is consistent with previous studies showing the additive effect on CFTR function rescue with three compounds with complementing working mechanisms (Veit et al., 2020). In 19 organoid cultures, AUC values of over 2000 were observed with the ABBV/GLPG-Triple. Comparable AUC values are obtained with VX-809/VX-770 and F508del/F508del organoids [around 2500 AUC, (Dekkers et al., 2016)], indicating that clinical efficacy of the ABBV/GLPG-Triple for pwCF compound heterozygous for F508del might be similar to the clinical effect of VX-809/VX-770 for pwCF homozygous for F508del-CFTR.

Interestingly, despite this general high efficacy, we observed great variation in response between both F508del/minimal function as well as between the F508del/F508del organoid cultures. The latter observation might indicate the presence of other *cis*-mutations in the CFTR gene or other genetic modifiers affecting CFTR-mediated fluid transport, two processes summarized in an excellent recent review (Paranjapye et al., 2020). Another possible explanation for the variation in drug efficacy between the F508del/minimal function organoids might be partial rescue of the minimal function allele with the ABBV/GLPG-modulators. We observed for example in the F508del/W1282X organoid culture some swelling response when pretreated with only one corrector, and hardly a difference between the dual ABBV/GLPG combinations and the triple ABBV/GLPG combination. This could be explained by the fact that the premature termination codon (PTC) mutation occurs late in the CFTR gene, resulting in only a minor truncation of the W1282X CFTR variant allowing further rescue by CFTR modulators, such as VX-770 as earlier described by (Berkers et al., 2019). This illustrates the potential contribution of the compound heterozygous mutations to the observed differences. In order to assess whether the variation in drug efficacy among pwCF is the result of rescue of the minimal function allele, future drug efficacy studies should also include allele specific biochemical analysis. Additional to the observed variation, two F508del/minimal function organoid cultures (expressing F508del/W846X and F508del/44374+1G > T CFTR) hardly responded to either VX-770/VX-809 or ABBV/GLPG-Triple, which corresponds to the results obtained with the VX-659/VX-661/VX-770 study, also showing two study participants not improving in mean change in FEV1 upon VX-659/VX-661/VX-770 therapy (Davies et al., 2018). Despite the overall variation, response to VX-770/VX-809 correlated in a linear positive manner with response to the ABBV/GLPG-Triple. This especially underlines the benefit of ABBV/GLPG-Triple therapy for patients that are mildly responsive to VX-770/VX-809.

Whether the ABBV/GLPG-Triple therapy is specifically effective for pwCF currently not benefitting from VX-445/VX-661/VX-770 therapy remains unclear, as the clinical trial reports did not share genotypes (Middleton et al., 2019). Future research should therefore investigate efficacy of the VX-445/VX-661/VX-770 in a large panel of F508del/minimal function organoids. In addition, it would be interesting to compare these results to the results obtained with the ABBV/GLPG-Triple. In future studies, it could be interesting to assess the efficacy of the GLPG/ABBV compounds in nasal/bronchial epithelial cells to confirm that rescue of CFTR is also achievable in airway-derived primary cells. Intestinal organoids are however superior over nasal or bronchial epithelial organoids when assessing function of CFTR by means of organoid swelling, as swelling of intestinal organoids is completely CFTR dependent whilst additional ion channels present in airway organoids also influence swelling. The effect of CFTR-mediated airway organoid swelling could therefore be underestimated. Finally, it should be noted that the absolute swelling values obtained in this manuscript cannot be directly compared to other published drug-induced FIS data, as experiments were performed with 5 µM or 0.8 µM forskolin instead of 0.128 µM forskolin (Dekkers et al., 2013; Berkers et al., 2019).

In summary, we confirm that combining compounds with complementary working mechanisms is a valuable approach for restoring CFTR function. We also show that characterizing compound efficacy in a personalized manner is required, as we observe great variation in drug efficacy between organoid cultures with comparable and even identical genotypes. Identifying individuals with a high modulator-responsive genotype ultimately will help in better understanding which genetic or cellular processes influence response to therapy and in defining personalized treatment regimes.

## Materials and Methods

### Collection of Primary Epithelial Cells

All experimentation using human tissues described herein was approved by the medical ethical committee at University Medical Center Utrecht (UMCU; TcBio#14-008) and performed following the guidelines of the European Network of Research Ethics Committees (EUREC) following European, national, and local law. Informed consent for tissue collection, generation, storage, and use of the organoids was obtained from all study participants. Biobanked intestinal organoids are stored and catalogued at the foundation Hubrecht Organoid Technology (HUB, http://hub4organoids.eu).

### Organoid Culture and FIS Assay

Crypts were isolated from rectal biopsies of subjects with cystic fibrosis as previously described (Dekkers et al., 2013). In brief, biopsies were washed with cold DMEM/F12 and incubated with 10 mM EDTA for 30 min. After harvesting the crypts containing supernatant, EDTA was washed away and crypts were seeded in 50% Matrigel in 24-well plates (∼10–30 crypts in three 10-ml Matrigel droplets per well). Growth medium (Paranjapye et al., 2020) was further supplemented with Primocin (1:500; Invivogen). Organoids were incubated in a humidified chamber with 5% CO_2_ at 37°C. Medium was refreshed every 2–3 days, and organoids were passaged 1:4 every 7 days. Prior to conducting FIS assays, organoids were cultured at least 3 weeks after thawing or crypt isolation. To quantify the organoid size increase over time, organoids are stained with calcein green (10 μM) which is added 30 min prior to the addition of forskolin and CFTR potentiators ABBV/GLPG-2451 and VX-770. All CFTR correctors ABBV/GLPG-2222 and GLPG/ABBV-2737 were added during plating of the organoids, 24 h prior to the FIS-assay. Forskolin was used at a 5 μM concentration, except in the FIS assay on F508del/F508del donors in which forskolin was used at 0.8 μM. All CFTR modulators were used at a 3 μM, with the exception of the FIS assay on F508del/F508del donors in which all ABBV/GLPG2451 and GLPLG/ABBV-2737 were used at 1 μM and ABBV/GLPG-2222 at 0.15 μM. Organoid swelling was measured using a confocal microscope, followed by quantification of total organoid surface area per well based on calcein staining. To correct for well to well differences in total organoid area, increase of organoid surface area over time was normalized to the organoid surface area of the first time point for each well as described by (Dekkers et al., 2013). All experiments with GLPG/ABBV compounds were conducted with two to three technical replicates. Orkambi stimulated FIS served as a positive control, while FIS with only forskolin served as negative control.

## Data Availability

The raw data supporting the conclusion of this article will be made available by the authors, without undue reservation.
